# Mortality-related risk factors of COVID-19: a systematic review and meta-analysis of 42 studies and 423,117 patients

**DOI:** 10.1186/s12879-021-06536-3

**Published:** 2021-08-21

**Authors:** Zelalem G. Dessie, Temesgen Zewotir

**Affiliations:** 1 School of Mathematics, Statistics and Computer Science, University of KwaZulu-Natal, Durban, South Africa; 2 College of Science, Bahir Dar University, Bahir Dar, Ethiopia

**Keywords:** Comorbidities, Demographic characteristics, Funnel plot, Heterogeneity, Publication bias, Sensitivity analysis

## Abstract

**Background:**

Mortality rates of coronavirus disease-2019 (COVID-19) continue to rise across the world. The impact of several risk factors on coronavirus mortality has been previously reported in several meta‐analyses limited by small sample sizes. In this systematic review, we aimed to summarize available findings on the association between comorbidities, complications, smoking status, obesity, gender, age and D-dimer, and risk of mortality from COVID-19 using a large dataset from a number of studies.

**Method:**

Electronic databases including Google Scholar, Cochrane Library, Web of Sciences (WOS), EMBASE, Medline/PubMed, COVID-19 Research Database, and Scopus, were systematically searched till 31 August 2020. We included all human studies regardless of language, publication date or region. Forty-two studies with a total of 423,117 patients met the inclusion criteria. To pool the estimate, a mixed-effect model was used. Moreover, publication bias and sensitivity analysis were evaluated.

**Results:**

The findings of the included studies were consistent in stating the contribution of comorbidities, gender, age, smoking status, obesity, acute kidney injury, and D-dimer as a risk factor to increase the requirement for advanced medical care. The analysis results showed that the pooled prevalence of mortality among hospitalized patients with COVID-19 was 17.62% (95% CI 14.26–21.57%, 42 studies and 423,117 patients). Older age has shown increased risk of mortality due to coronavirus and the pooled odds ratio (pOR) and hazard ratio (pHR) were 2.61 (95% CI 1.75–3.47) and 1.31 (95% CI 1.11–1.51), respectively. A significant association were found between COVID-19 mortality and male (pOR = 1.45; 95% CI 1.41–1.51; pHR = 1.24; 95% CI 1.07–1.41), and current smoker (pOR = 1.42; 95% CI 1.01–1.83). Furthermore, risk of mortality among hospitalized COVID-19 patients is highly influenced by patients with Chronic Obstructive Pulmonary Disease (COPD), Cardiovascular Disease (CVD), diabetes, hypertension, obese, cancer, acute kidney injury and increase D-dimer.

**Conclusion:**

Chronic comorbidities, complications, and demographic variables including acute kidney injury, COPD, diabetes, hypertension, CVD, cancer, increased D-dimer, male gender, older age, current smoker, and obesity are clinical risk factors for a fatal outcome associated with coronavirus. The findings could be used for disease’s future research, control and prevention.

## Introduction

The 2019 novel coronavirus (2019-nCoV) is a newly emerging disease that was first reported in China, and has subsequently spread worldwide. COVID-19 is caused by severe acute respiratory syndrome coronavirus 2 (SARS-CoV-2), which belong to the family of Betacoronavirus genus [1]. Although the clinical presentation and symptoms of COVID-19 are similar to that of Middle East Respiratory Syndrome (MERS) and Severe Acute Respiratory Syndrome (SARS), the rate of spread is greater [2]. On 11 March 2019, the WHO defined COVID-19 as a pandemic disease [3], and as of February 2021, a total of 107,496,792 cases and 2,353,308 deaths (3.0%) have been confirmed worldwide in 219 countries [4]. It is a major challenge for many countries to identify what measures could be used to avoid death or severe illness.

The challenge of COVID-19 is very high globally due to the complexity of its transmission and a lack of proven treatment [5, 6]. It will be more disastrous for middle and low-income countries because of their high illiteracy, a very poor health care system, and a scarce Intensive Care Unit. A series of studies have reported clinical characteristics of COVID-19 critical illness [7] and severe illness [8] patients. The clinical features and risk factors considered aims for the identification of risk factors associated with fatal outcomes. Regardless of the scientist’s effort to better understand the diagnostic, and clinical characteristics of the disease, our current understanding of patient's risk factors of death with COVID-19 is still limited. Accordingly one might not exhaustively study all possible risk factors. In every study, the considered risk factors vary in number and type. Based on the literature review we studied the commonly reported risk factors such as hypertension, diabetes, chronic obstructive pulmonary disease, dyspnoea, history of substance use, gender, acute respiratory distress syndrome (ARDS), history of smoking, older age, albumin, and D-dimer [9–12]. The study aims to synthesize and enhance our understanding about the precision of the risk factors effect on COVID-19 fatality rate.

## Methods

### Study protocol

To examine the association between COVID-19 mortality versus in with comorbidities, gender, smoking status, obesity, age, acute kidney injury, and D-dimer, we followed PRISMA guidelines [13] to perform the meta-analysis of the articles identified through our systematic reviews.

### Search strategy

Electronic databases including Google Scholar, Cochrane Library, Web of Sciences (WOS), EMBASE, Medline/PubMed, COVID-19 Research Database (WHO), COVID-19 Open Research Dataset Challenge, and Scopus, were systematically searched till 31 August 2020. The search strategy was as follows: (“severe acute respiratory syndrome coronavirus 2” or “novel coronavirus” or “COVID-19” or “2019-nCoV” or “SARS-CoV-2”) and (“survival” or “fatal outcome” or “mortality” or “death”). Furthermore, the search was specifically focused on articles that analyzed laboratory parameters, pre-existing comorbidities, clinical status, and demographic characteristics as potential predictors for fatal outcome of COVID-19. No restriction was applied on time and language of publications. In order to improve the screening process and save time, we downloaded the literature results into EndNote X9.

### Eligibility criteria

**Fig. 1:**
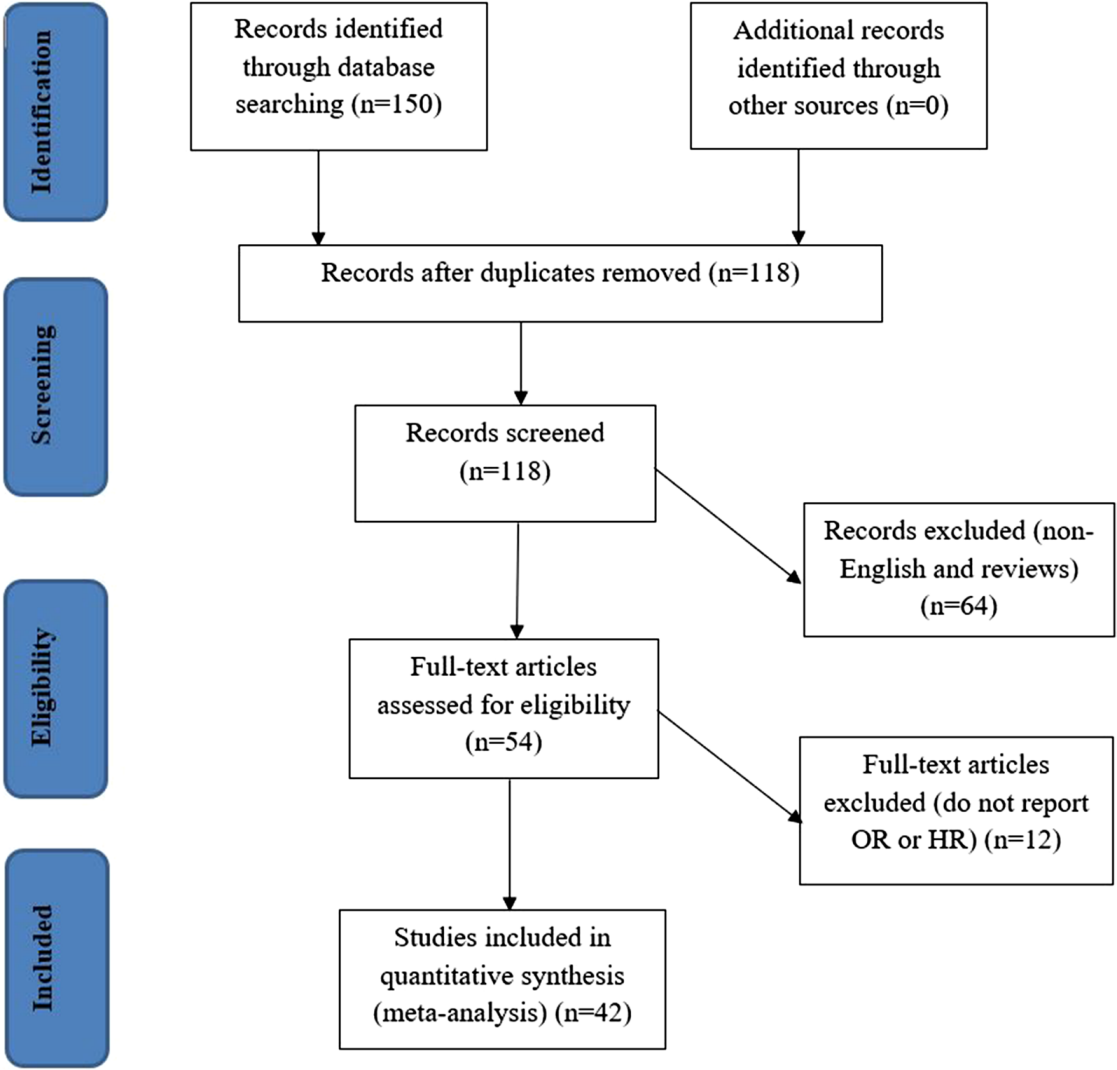
Flow chart of the study search and screening process

Once duplicates were removed, the initial search results were screened for relevance by titles and abstracts by both authors. The full texts were reviewed for the eligibility criteria (Fig. 1). Studies without abstract and/or full text, Correspondence letters, COVID-19 studies on children only, editorials, reviews, qualitative studies, books, theses, expert opinion papers, and review articles were excluded from the analysis. Furthermore, among the eligible studies, we used if only the study reported odds ratios (ORs) or hazard ratios (HRs) along with 95% CI for the association between demographic or epidemiological or clinical characteristics and fatal outcome of coronavirus.

### Data extraction and assessment for study quality

Both authors independently examined the downloaded EndNote X9 search outputs eligibility for inclusion. Any disagreements between the authors were resolved through discussion and mutual agreement. Both authors extracted the following data: the first author’s name, countries, assessment methods, sample size, study design, the publication year, demographic variables (e.g., gender, age, etc.), clinical variables (e.g., comorbidities, complications, D-dimer, etc.), outcome (mortality), exposure (risk factors), and adjusted odds ratios or hazard ratios or relative risk. The authors independently evaluated the quality methodological approach of the articles using a Newcastle–Ottawa technique [14]. In this technique, three main components were utilized to assess the quality of the papers such as assessment of the outcome, comparability of the study groups, and selection procedure of the study patients. The Newcastle–Ottawa technique included seven domains, each one of these domains were scored from 3 to 0 (i.e., from low to high bias) and their average score were taken.

### Statistical analysis

We have used peer-reviewed and published ORs or HRs (and their 95%CI) for the association between the fatal outcome of COVID-19 and risk factors. A mixed-effect model has been computed keeping into consideration the expected between-study heterogeneity. Heterogeneity in effect sizes was assessed by computing Cochran’s Q test; a significant *Q* indicates a lack of homogeneity and inference of heterogeneity. The proportion of the total variance attributable to the study heterogeneity was determined using *I*^2^ statistic [15]. The *I* ^2^ values of 60–90%, 40–59%, and 0–39% were considered to indicate severe, moderate, and mild, respectively [15]. Funnel plots with Egger weighted regression test were used for assessing publication bias [16]. All of the analyses were implemented with the statistical software’s R-4.0.2 and STATA version 16, to estimate the pooled odds ratio and to investigate publication bias.

## Results

### Search results

We identified 150 publications through Google Scholar, Cochrane Library, Web of Sciences (WOS), EMBASE, Medline/PubMed, COVID-19 research database (WHO), COVID-19 open research dataset challenge, and Scopus database, of which, 14 studies that did not have numbers of hospital death, 31 reviews, 19 non-English, and 32 duplicates were excluded. Among the remaining 54 studies, twelve did not report cross-tabulation with ORs or HRs. Consequently, we got only 42 studies that satisfied all the eligibility criteria (see Fig. 1). Out of the 42 studies, thirty-nine provided adjusted hazard and odds ratios after multivariate adjustment for the covariates such as comorbidities, gender, smoking status, obesity, age, acute kidney injury, and D-dimer [17–52]. And the rest three studies provided crude hazard and odds ratios [53–55] (Table 1).

**Table 1:** Characteristics of studies included in the systematic review and meta-analysis on the effect of comorbidities, gender, age, smoking status, obesity, acute kidney injury, and D-dimer gender on fatal outcome of COVID-19

Authors (year)	Country	Sample size	Death	Mean (± SD) /Median [IQR] of age	Males N (%)	CVD N (%)	DM N (%)	HT N (%)	COPD N (%)	Cancer N (%)	OR or HR (95%CI)
Albitar et al. [51]	Asian	828	219	49.4 (20.9) ^a^	489 (59.1)	23 (2.8)	62 (7.5)	90 (10.9)	NA	NA	Age: Old: aOR = 1.08 (1.06–1.09) Sex: Male: aOR = 1.61 (1.01–2.58) HT: Yes: aOR = 3.58(1.69–7.55)
Al‐Salameh et al. [52]	France	432	89	72.0 (14.3) ^a^	238 (55.1)	148 (34.3)	115 (26.6)	255 (59.0)	39 (9.02)	NA	Obese: Yes: aOR = 1.78 (1.06–3.00) Cardiac disease: Yes: aOR = 2.01 (1.13–3.58)
Barman et al. [50]	Turkey	607	103	69.3 (12.5) ^a^	334 (55.0)	NA	192 (31.6)	266 (43.8)	73 (12.0)	NA	Age: Old: aOR = 1.03 (1.01–1.05) HT: Yes: aOR = 1.26 (0.60–2.62) DM: Yes: aOR = 1.39 (0.89–2.17) Cardiac injury: Yes: aOR = 10.58 (2.42–46.27)
Berenguer et al. [49]	Spain	4035	1131	70 [56–80]	2433 (61.0)	932 (23.3)	871 (21.8)	2052 (51.2)	715 (17.9)	NA	Age: Old: aHR = 2.72 (1.74–4.23) Sex: Male: aHR = 1.29 (1.13–1.49) HT: Yes: aHR = 1.22 (1.05–1.40) Obese: Yes: aHR = 1.53 (1.28–1.84) Cancer: Yes: aHR = 1.49 (1.24–1.79)
Caliskan and Saylan [82]	Turkey	565	75	48 (19.67)	NA	NA	72 (12.7)	128 (22.7)	37 (6.5)	NA	Age: Old: aOR = 1.05 (1.03–1.11) Smoking: Yes: aHR = 6.51 (2.73–15.5) COPD: Yes: aOR = 3.21 (1.22–8.43)
Chen et al. [46]	China	1590	50	69 [51–86]	904 (56.8)	30 (1.9)	130 (8.2)	269 (16.9)	24 (1.5)	NA	Age: Old: aHR = 3.43 (1.24–9.50) CVD: Yes: aHR = 3.10 (1.07–8.94)
Chen et al. [47]	China	1859	208	59 [45–68]	925 (50)	NA	262 (14)	579 (31)	61 (3)	69 (4)	Age: Old: aHR = 1.04 (1.03–1.06) Smoking: Yes: aHR = 1.84 (1.17–2.92) D-dimer: High: aHR = 3.00 (2.17–4.16)
Chen et al. [48]	China	3309	307	62 [51–69]	1642 (51.5)	242 (7.3)	464 (14)	988 (29.9)	42 (1.3)	NA	Age: Old: aOR = 9.08 (4.44–18.59) Sex: Female: aOR = 0.44 (0.34–0.58) HT: Yes: aOR = 1.14(0.87–1.50) DM: Yes: OR = 0.87(0.70–1.36) CVD: Yes: aOR = 1.41(0.94–2.13) COPD: Yes: aOR = 1.72(0.80–3.71) Kidney disease: Yes: aOR = 2.85(1.42–5.73)
Chilimuri et al. [45]	USA	375	160	63 [52–72]	236 (63)	62 (17)	175 (47)	225 (60)	NA	NA	Age: Old: aOR = 1.04 (1.01–1.06) CVD: Yes: aOR = 1.56 (0.78–3.11) HT: Yes: aOR = 2.43 (1.57–3.77) DM: Yes: aOR = 1.96 (1.29–2.98) D-dimer: High: aOR = 3.16 (1.75–5.73)
Colombi et al. [55]	Italy	236	108	68 [66–70]	177 (75)	127 (54)	37 (16)	NA	NA	35 (15)	Age: Old: aOR = 3.4 (1.7–6.6) Cancer: Yes: aOR = 3.5 (1.6–7.7) CVD: Yes: aOR = 3.7 (1.9–7.3)
Cummings et al. [71]	USA	257	101	62 [51–72]	171 (67)	49 (19)	92 (36)	162 (63)	24 (9)	NA	Age: Old: aHR = 1.31 (1.09–1.57) Sex: Male: aHR = 1.13 (0.71–1.81) HT: Yes: aHR = 1.58 (0.89–2.81) DM: Yes: aHR = 1.31(0.81–2.10) COPD: Yes: aHR = 2.94 (1.48–5.84) Increase D-dimer: aHR = 1.10 (1.01–1.19) Cardiac disease: Yes: aHR = 1.76 (1.08–2.86)
Du et al. [44]	China	179	21	57.6 (13.7) ^a^	97 (54.2)	29 (16.2)	33 (18.4)	58 (32.4)	NA	2 (2.2)	Age: Old: aOR = 3.77 (1.20–11.80) CVD: Yes: aOR = 2.46 (1.28–4.75) Cardiac disease: Yes: aOR = 4.07 (1.78–9.35)
Fabio et al. [43]	Italy	410	95	65 [56–75]	299 (72.9)	51 (12.6)	69 (17.0)	203 (49.9)	22 (5.4)	22 (5.4)	Age: Old: aHR = 3.17 (1.84–5.44) Cancer: Yes: aHR = 2.32 (1.15–4.64) DM: Yes: aHR = 1.51 (0.96–2.05)
Grasselli et al. [42]	Italy	3988	1926	63 [55–69]	3188 (79.9)	538 (13.5)	514 (12.9)	1643 (41.2)	93 (2.3)	331 (8.3)	Age: Old: aHR = 1.75 (1.60–1.92) Sex: Men: aHR = 1.57 (1.31–1.88) HT: Yes: aHR = 0.99 (0.81–1.22) DM: Yes: aHR = 1.18 (1.01–1.39) COPD: Yes: aHR = 1.68 (1.28–2.19)
Guan et al. [41]	China	1590	131	48.9 (16.3) ^a^	904 (57.3)	59 (3.7)	130 (8.2)	269 (16.9)	24 (1.5)	18 (1.1)	HT: Yes: aHR = 1.58 (1.07–2.32) DM: Yes: aHR = 1.59 (1.03–2.45) COPD: Yes: aHR = 2.68 (1.42–5.05) Cancer: Yes: aHR = 3.50 (1.60–7.64)
Hernández-Galdamez et al. [40]	Mexico	211,003	7135	45.7 (16.3) ^a^	115,442 (54.7)	4949 (2.35)	34,685 (16.4)	42,453 (20.1)	3721 (1.8)	NA	DM: Yes: aOR = 1.69 (1.63–1.74) COPD: Yes: aOR = 1.20 (1.11–1.30) Obese: Yes: aOR = 1.42 (1.37–1.47) Kidney disease: Yes: aOR = 2.31 (2.15–2.48) HT: Yes: aOR = 1.24 (1.20–1.28)
Klang et al. [39]	USA	3406	1076	76.0 [67–84]	1961 (57.6)	513 (15.1)	1599 (46.9)	2299 (67.5)	NA	530 (15.6)	Age: Old: aOR = 1.70 (1.60–1.80) Sex: Male: aOR = 1.40 (1.20–1.60) Obese: Yes: aOR = 1.60 (1.20–2.30) DM: Yes: aOR = 1.40 (1.20–1.70) Kidney disease: Yes: aOR = 1.70 (1.20–2.10)
Kuderer et al. [38]	USA	928	121	66 [57–76]	468 (50.4)	NA	NA	NA	NA	294 (31.7)	Age: Old: aOR = 1.84 (1.53–2.21) Sex: Male: aOR = 1.63 (1.07–2.48) Smoking: Yes: aOR = 1.60 (1.03–2.47) Cancer: Yes: aOR = 1.79 (1.09–2.95)
Lee et al. [37]	Korea	98	20	72 [68–79]	44 (44.9)	16 (16.3)	27 (27.6)	52 (53.1)	NA	11 (11.2)	Sex: Male: aOR = 3.70 (1.29–11.11) HT: Yes: aOR = 1.95 (1.56–2.80) DM: Yes: aOR = 4.74 (1.68–13.38)
Lee et al.[36]	UK	800	226	69 [59–76]	449 (56)	109 (14)	131 (16)	247 (31)	61 (8)	NA	Age: Old: aOR = 9.42 (6.56–10.02) Sex: Male: aOR = 1.67 (1.19–2.34) HT: Yes: aOR = 1.95 (1.36–2.80) DM: Yes: aOR = 1.61 (1.03–2.48) CVD: Yes: aOR = 2.32 (1.47–3.64) COPD: Yes: aOR = 1.80 (1.01–3.27)
Li et al. [35]	China	548	87	60 [48–69]	276 (50.9)	34 (6.2)	83 (15.1)	166 (30.3)	17 (3.1)	24 (4.7)	Age: Old: aHR = 1.72 (1.05–2.85) Sex: Male: aHR = 1.72 (1.09–2.73) Cardiac injury: Yes: aHR = 2.92 (1.80–4.76)
Lim et al. [34]	Korea	160	44	67 [24–92]	86 (53.8)	21 (13.1)	50 (31.3)	78 (48.1)	NA	NA	Age: Old: aHR = 1.04 (1.01–1.07) Kidney disease: Yes: aHR = 3.62 (1.75–7.48) Sex: Male: aHR = 0.61 (0.32–1.16) HT: Yes: aHR = 1.34 (0.71–2.52) DM: Yes: aHR = 1.35 (0.72–2.56)
Mehra et al. [33]	North America, Asia and Europe	8910	515	49.0 (16.0) ^a^	5339 (59.9)	NA	1272 (14.3)	2349 (26.3)	225 (2.5)	NA	Age: Old: aOR = 1.93 (1.60–2.41) Sex: Male: aOR = 1.26 (1.05–1.54) COPD: Yes: aOR = 2.96 (2.00–4.40) Smoking: Yes: aOR = 1.79 (1.29–2.47) CVD: Yes: aOR = 2.48 (1.62–2.47)
Mikami et al. [32]	USA	6493	858	59 [43, 72]	3538 (54.5)	NA	1151 (17.7)	1637 (25.2)	176 (2.7)	413 (6.4)	Age: Old: aHR = 4.85 (2.75–8.56) Sex: Male: aHR = 1.22 (1.11–1.33) HT: Yes: aHR = 0.91 (0.79–1.07) DM: Yes: aHR = 0.92 (0.73–1.16) Cancer: Yes: aHR = 1.08 (0.84–1.40) D-dimer: High: aHR = 1.19 (1.02–1.39)
Palaiodimos et al. [31]	USA	200	48	64 [50–74]	98 (49)	22 (11)	79 (39.5)	152 (76)	28 (14)	NA	Age: Old: aOR = 1.73 (1.25–5.98) Sex: Male: aOR = 2.74 (1.25–5.93) DM: Yes: aOR = 1.16 (0.55–2.44) BMI: Obese: aOR = 3.78 (1.45–9.83) COPD: Yes: aOR = 2.05 (0.76–5.51)
Parra-Bracamonte et al. [30]	Mexico	142,690	16,872	44 [33–56]	79,280 (56)	NA	23,803 (17)	28,874 (20)	2655 (2)	NA	Age: Old: aOR = 3.73 (2.99–4.65) Sex: Male: aOR = 1.45 (1.39–1.50) HT: Yes: aOR = 1.24 (1.19–1.29) DM: Yes: aOR = 1.28 (1.24–1.34) COPD: Yes: aOR = 1.26 (1.15–1.38) Obese: Yes: aOR = 1.23 (1.17–1.28) Kidney disease: Yes: aOR = 1.8 (1.66–1.96)
Petrilli et al. [29]	USA	5279	665	54 [38–66]	2615 (49.5)	2752 (52.1)	1195 (22.6)	2256 (42.7)	786 (14.9)	403 (7.6)	Age: Old: aHR = 7.69 (4.60–12.84) Sex: Male: aHR = 1.27 (1.08–1.50) HT: Yes: aHR = 0.94 (0.76–1.16) Obese: Yes: aHR = 1.41 (0.98–2.01) DM: Yes: aHR = 1.10 (0.93–1.31) COPD: Yes: aHR = 0.93 (0.76–1.15) Cancer: Yes: aHR = 1.31 (1.05–1.62) Kidney disease: Yes: aHR = 1.18 (0.97–1.43)
Pettit et al. [28]	USA	238	24	58.5 (17.0) ^a^	113 (47.5)	51 (21.4)	68 (28.6)	126 (52.9)	NA	27 (11.3)	Age: Old: aOR = 3.6 (2.0–6.3) Obese: Yes: aOR = 1.7 (1.1–2.8)
Price-Haywood et al. [27]	Australia	3481	326	55.5 (18.5) ^a^	1394 (40.0)	NA	566 (16.3)	1074 (30.8)	79 (2.3)	158 (4.5)	Age: Old: aOR = 1.19 (1.13–1.24) Sex: Male: aOR = 1.61 (1.28–2.04) Obese: Yes: aOR = 1.05 (0.83–1.34)
Priyank et al. [26]	USA	522	92	63 [50–72]	218 (41.8)	70 (13.4)	221 (42.3)	416 (79.7)	47 (9)	48 (9.2)	Age: Old: aOR = 3.1 (1.7–5.6) Sex: Male: aOR = 2.44 (1.43–4.17) HT: Yes: aOR = 3.36 (1.3–8.6) DM: Yes: aOR = 1.51 (0.9–2.6) COPD: Yes: aOR = 1.48 (0.65–3.34) Kidney disease: Yes: aOR = 1.08 (0.51–2.28) Cancer: Yes: aOR = 0.48 (0.20–1.10)
Regalado-Artamendi et al. [74]	Spain	177	61	70 [56–77]	99 (55.9)	NA	33 (18.6)	73 (41.2)	NA	NA	Age: Old: aHR = 1.05 (1.03–1.07) Sex: Male: aHR = 1.07 (0.65–1.77) HT: Yes: aHR = 1.79 (1.09–2.96) DM: Yes: aHR = 1.13 (0.60–2.10) Kidney disease: Yes: aHR = 2.36 (1.04–5.38) D-dimer: High: aOR = 1.26 (1.01–1.56)
Rivera-Izquierdo et al. [25]	Spain	238	61	64.7 (15.4)^a^	131 (55.0)	54 (22.7)	52 (21.9)	116 (48.7)	NA	NA	Age: Old: aHR = 1.09 (1.07–1.11) Sex: Male: aHR = 1.34 (0.80–2.27) DM: Yes: aHR = 2.33 (1.38–3.94)
Shi et al. [23]	China	416	57	64 [21–95]	205 (49.3)	44 (10.6)	60 (14.4)	127 (30.5)	12 (2.9)	9 (2.2)	Age: Old: aHR = 1.02 (0.99–1.05) CVD: Yes: aHR = 1.51 (0.70–3.30) COPD: Yes: aHR = 0.37 (0.04–3.50) Cardiac disease: Yes: aHR = 4.26 (1.92–9.49)
Soares et al. [22]	Brazil	10,713	821	NA	4804 (44.8)	2541 (23.7)	1100 (10.3)	NA	NA	NA	Age: Old: aOR = 3.95 (2.95–5.33) CVD: Yes: aOR = 2.02 (1.59–2.57) DM: Yes: aOR = 1.68 (1.10–3.09)
Su et al. [54]	China	172	32	71.6 (11.0) ^a^	121 (70.3)	21 (12.2)	18 (10.5)	18 (11)	6 (3.4)	3 (1.7)	HT: Yes: OR = 3.5 (1.1–10.8) DM: Yes: OR = 1.9 (0.6–5.5) Sex: Male: OR = 1.53 (0.75–3.13) CVD: Yes: OR = 5.1 (1.7–15.5)
van Gerwen et al. [21]	USA	3703	616	56.8 (18.2) ^a^	2049 (55.3)	292 (7.9)	1045 (28.2)	1643 (44.4)	172 (4.6)	312 (8.4)	Age: Old: aOR = 5.29 (2.51–11.15) Sex: Male: aOR = 1.46 (1.17–1.82) HT: Yes: aOR = 1.87 (1.53–2.29) DM: Yes: aOR = 1.62 (1.34–1.96) CVD: Yes: aOR = 1.47 (1.06–2.02) Smoking: Yes: aOR = 1.06 (0.66–1.72)
Wang et al. [20]	China	339	65	69 [65–76]	166 (49)	53 (15.7)	54 (16)	138 (40.8)	21 (6.2)	15 (4.4)	Age: Old: aHR = 1.06 (1.03–1.09) CVD: Yes: aHR = 1.85 (1.06–3.26) COPD: Yes: aHR = 2.24 (1.12–4.97)
Wu et al. [53]	China	201	44	51 [43–60]	128 (64)	8 (4)	22 (11)	39 (19.4)	NA	1 (0.5)	Age: Old: HR = 6.17 (3.26–11.67) HT: Yes: HR = 1.70 (0.92–3.14) DM: Yes: HR = 1.58 (0.80–3.13) D-dimer: High: HR = 1.02 (1.01–1.04)
Xu et al. [19]	China	239	147	62.5 (13.3)^a^	143 (59.8)	NA	44 (18.4)	105 (43.9)	NA	NA	Age: Old: aHR = 1.57 (1.12–2.19) Cardiac injury: Yes: aHR = 0.88(0.57–1.34) Kidney disease: Yes: aHR = 2.06 (1.36–3.10)
Yao et al. [18]	China	248	17	63.0 (13.4)^a^	NA	NA	44 (17.7)	78 (31.5)	NA	NA	Age: Old: aOR = 1.04 (0.98–1.10) D-dimer: High: aOR = 10.17 (1.10–29.38)
Yu et al. [83]	China	1464	212	64 [51–71]	736 (51.3)	47 (3.2)	211 (14.4)	306 (20.9)	50 (3.4)	17 (1.2)	Sex: Male: aOR = 1.97 (1.29–2.99) Age: Old: aOR = 2.15 (1.35–3.43) HT: Yes: aOR = 1.08 (0.68–1.72) DM: Yes: aOR = 2.34 (1.45–3.76)
Zhou et al. [17]	China	191	54	56 [46–67]	119 (62)	15 (8)	36 (19)	58 (30)	6 (3)	NA	Age: Old: aOR = 1.10 (1.03–1.17) D-dimer: High: aOR = 18.42(2.64–29.39)

*aHR* adjusted hazard ratio, *aOR* adjusted odds ratio, *CVD* cerebrovascular disease, *HT* hypertension, *COPD* chronic obstructive pulmonary disease, *DM* diabetes, *COVID-19* coronavirus disease 2019, *IQR* interquartile range

^a^Reported as mean (± SD). Other studies were reported as median (IQR)

### Demographic characteristics and geographical distribution

Table 1 presents a systematic summary of all the selected studies [2, 6–9, 12, 15, 18–21, 25, 26, 28, 30, 35, 40, 42, 45–47, 50–52, 55–68]. All the 42 studies were published in the year 2020. All included studies were conducted in COVID-19 outbreak areas from December 2019 to August 2020. The studies reported a total of 423, 117 patients. Of these, 13 were performed in mainland China, 11 in USA, 2 in Spain, 2 in Mexico, 2 in Korea, 3 in Italy, 1 in France, 1 in Australia, 1 in Asia, 1 in Brazil, 1 in UK, 2 in Turkey and 2 mixed region. The sample size of enrolled patients ranged from 98 to 211,003 individuals. The proportions of male in the study samples ranged from 41.8 to 70.3%. The average age of individuals included in the studies ranged from 48.9 to 77 years. (Table 1).

### Prevalence of COVID-19 mortality

**Fig. 2:**
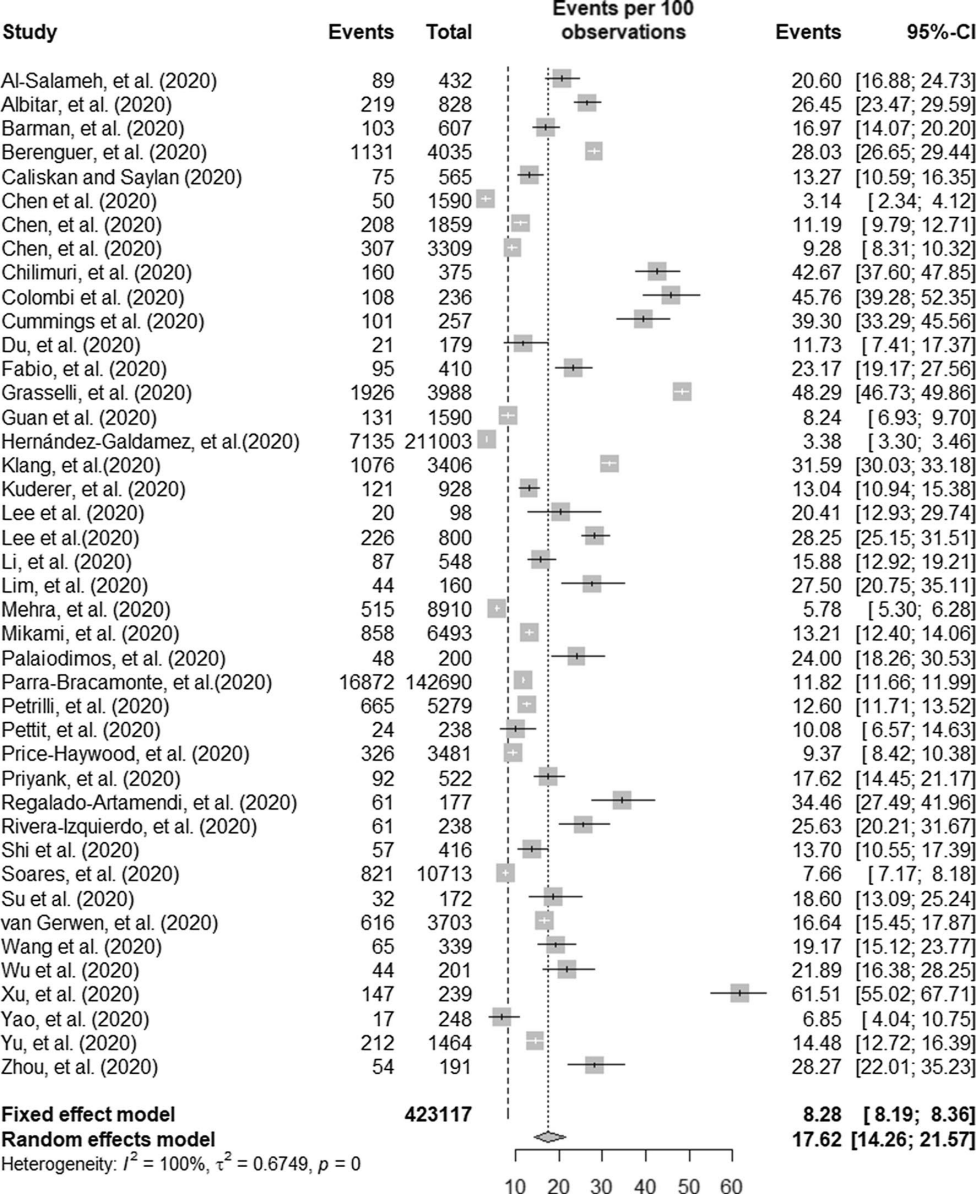
Forest plot showing the proportion of COVID-19 mortality

The mixed effect meta-analysis model results are presented in Fig. 2. From this plot, we can see that the mortality rate of coronaviruses among the included studies ranges from a minimum of 3.14 (95% CI 2.34–4.12%) [46] to a maximum of 61.51 (95% CI 55.02–67.71%) [19]. Of the total 423 117 patients, 35 020 died which resulted in a weighted pooled overall mortality prevalence of 17.62% (95% CI, 14.26–21.57%). (Fig. 2).

### Mortality-related risk factors

**Fig. 3:**
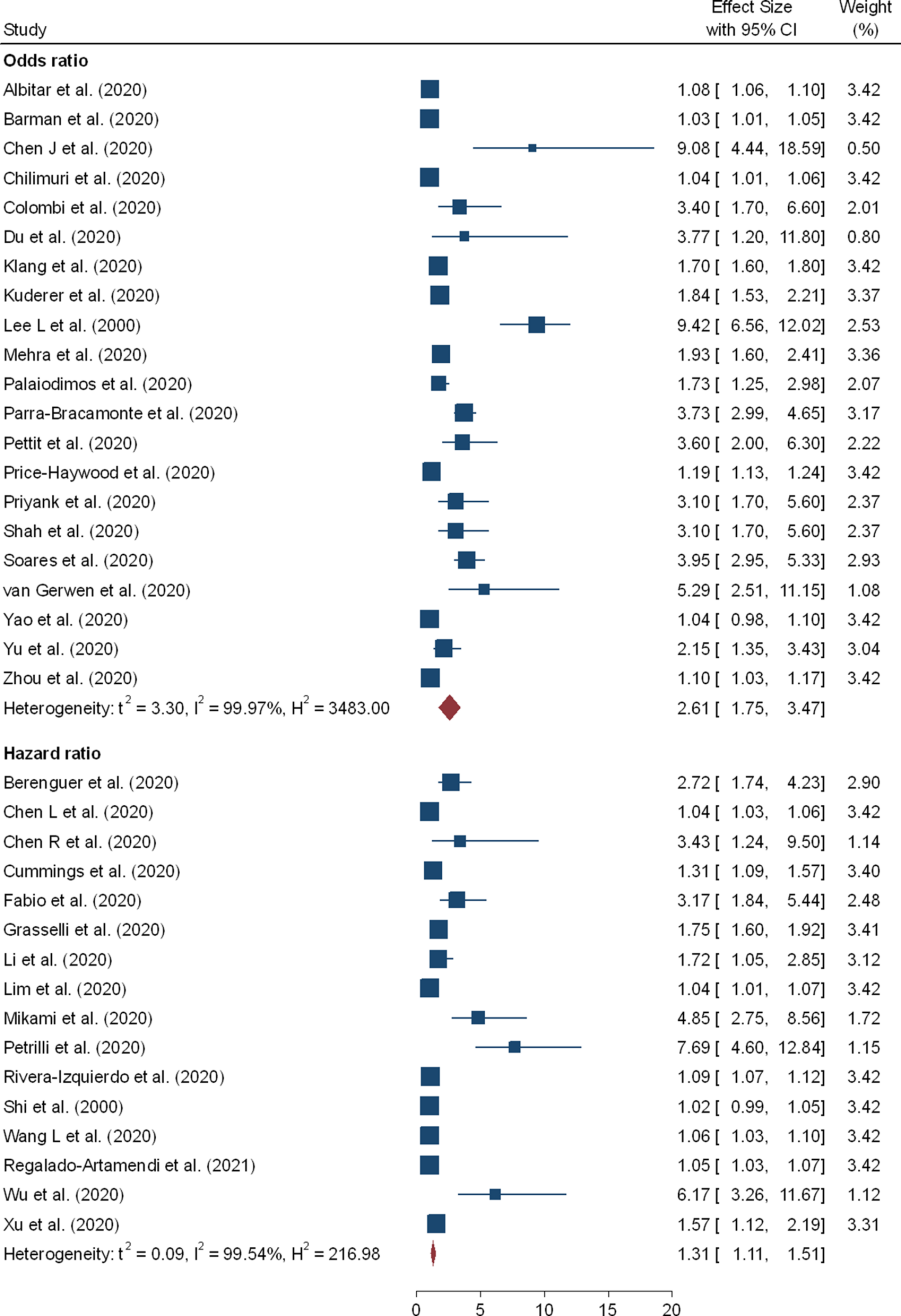
Forest plot showing the estimate for the effects of age on COVID-19 mortality

**Fig. 4:**
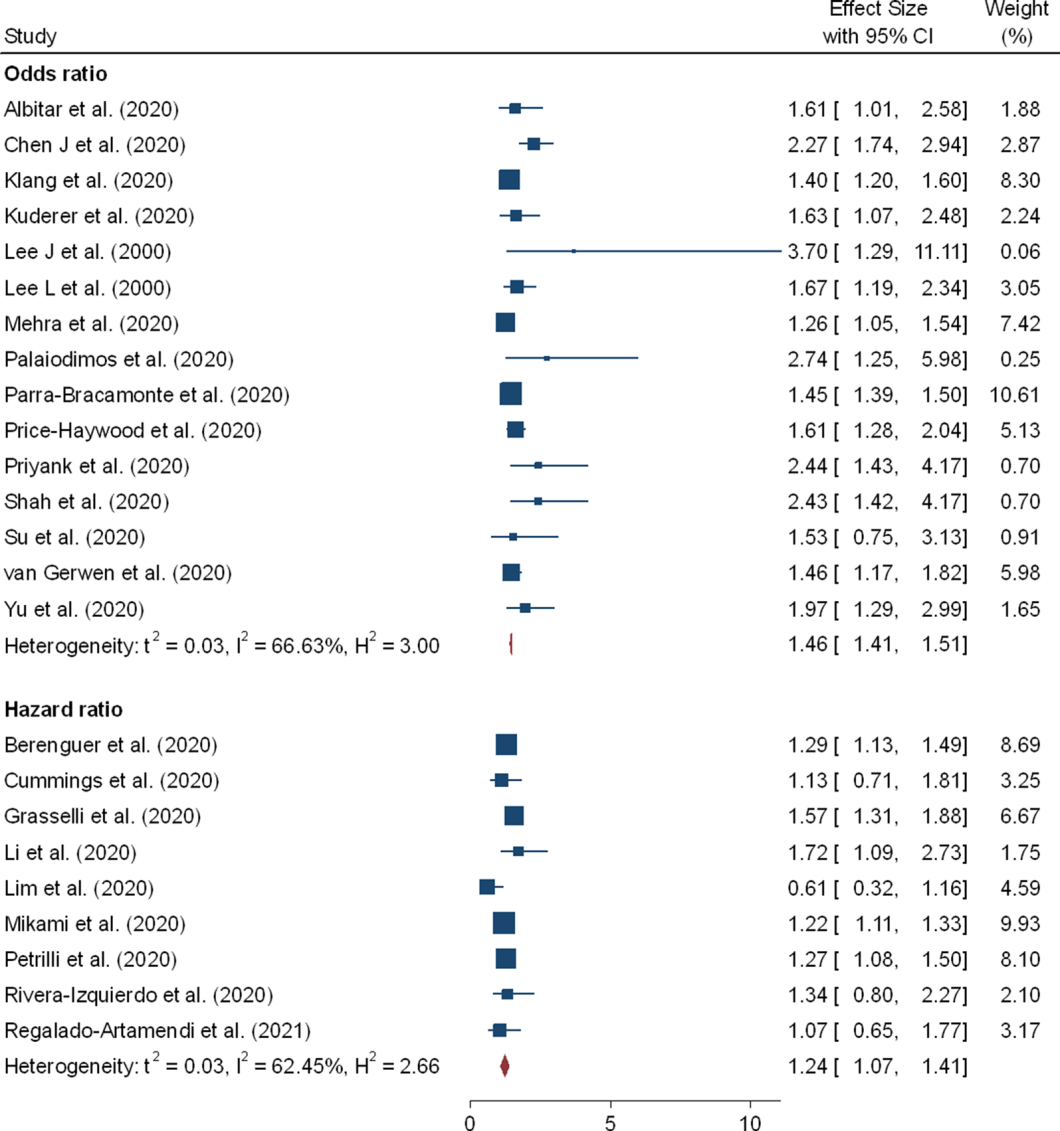
Forest plot showing the estimate for the effects of gender on COVID-19 mortality

**Fig. 5:**
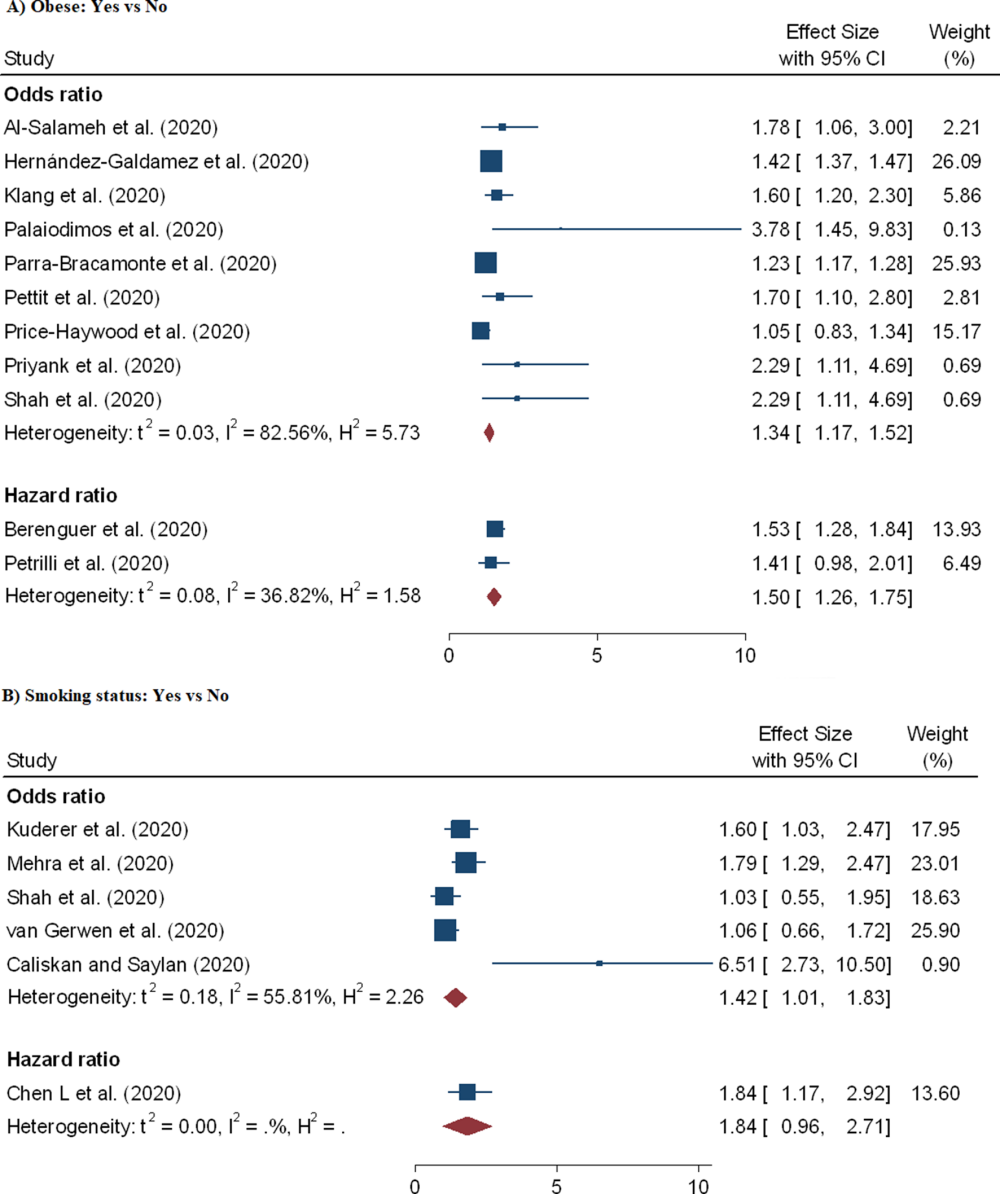
Forest plot showing the estimate for the effects of smoking status and obesity on COVID-19 mortality

In the meta-analysis 32 effect sizes of the demographic characteristics were obtained from 37 studies [5, 9–12, 15, 17, 18, 20–22, 28, 29, 38, 40, 47–51, 53, 55, 62, 66, 67, 69, 70] (26 162 cases of death out of 203 250 patients). Older age has shown increased risk of mortality due to coronavirus and the pooled OR and HR were 2.61 (95% CI 1.75–3.47) and 1.31 (95% CI 1.11–1.51), respectively (Table 2 and Fig. 3). Twenty-four studies evaluated the risk of COVID-19 mortality among male patients and showed a significantly higher risk and the pooled OR and HR were 1.45 (95% CI 1.41–1.51) and 1.24 (95% CI 1.07–1.41), respectively (Table 2 and Fig. 4). Coronavirus related risk of mortality was significantly associated with smoker patients when compared to non-smoker patients, (pOR = 1.42; 95%CI = 1.01–1.83) (Fig. 5B). Furthermore, the combined 11 effect sizes from 11 studies [2, 10, 21, 22, 24, 28, 31, 40, 44, 51, 56] revealed significant association between obesity and coronaviruses mortality (pOR = 1.34; 95%CI = 1.17–1.52; pHR = 1.50; 95%CI = 1.26–1.75) (Fig. 5A).

**Table 2:** Results of the subgroup analysis based on demographic and clinical variables associated with coronavirus mortality

Risk factors	Effect measures	Numbers of study	Effect size (95% CI)	Heterogeneity		Begg’s test P-value^#^	Egger’s test P-value^#^
				I^2^	P-value		
Older age	pOR	21	2.61 (1.75–3.47)	99.97	0.000	0.321	0.531
	pHR	16	1.31 (1.11–1.51)	99.54	0.000	0.212	0.142
Gender: Male vs Female	pOR	15	1.45 (1.41–1.51)	66.63	0.000	0.243	0.213
	pHR	9	1.24 (1.07–1.41)	62.45	0.000	0.424	0.126
Smoking status: Yes vs No	pOR	5	1.42 (1.01–1.83)	55.81	0.000	0.143	0.076
	pHR	1	1.84 (0.96–2.71)	–	–	–	–
Obesity: Yes vs No	pOR	9	1.34 (1.17–1.52)	82.56	0.000	0.293	0.272
	pHR	2	1.50 (1.26–1.75)	36.82	0.070	0.253	0.312
CVDs: Yes vs No	pOR	9	1.83 (1.50–2.17)	41.27	0.020	0.410	0.388
	pHR	3	1.77 (0.95–2.59)	13.73	0.160	0.426	0.143
Diabetes	pOR	13	1.52 (1.36–1.69)	79.83	0.000	0.432	0.471
	pHR	10	1.17 (1.02–1.32)	49.45	0.000	0.298	0.462
Hypertension	pOR	12	1.57 (1.27–1.87)	94.97	0.000	0.114	0.399
	pHR	8	1.18 (1.01–1.40)	66.66	0.000	0.054	0.267
COPD	pOR	7	1.58 (1.08–2.07)	92.24	0.000	0.130	0.146
	pHR	5	1.71 (1.01–2.45)	78.28	0.000	0.092	0.078
Cancer	pOR	3	1.43 (0.06–2.80)	79.98	0.000	0.181	0.162
	pHR	5	1.33 (1.09–1.56)	58.67	0.000	0.461	0.234
Acute kidney injury	pOR	5	1.87 (1.48–2.26)	86.53	0.000	0.131	0.220
	pHR	3	2.21 (1.44–2.99)	42.43	0.030	0.256	0.087
Cardiac injury	pOR	3	2.33 (0.88–3.79)	5.97	0.320	0.088	0.090
	pHR	4	1.89 (0.75–3.02	76.57	0.000	0.065	0.102
Increased D-dimer	pOR	3	10.49 (1.80–19.18)	96.14	0.000	0.312	0.101
	pHR	5	1.44 (1.01–2.06)	91.52	0.000	0.067	0.178

Keys: (#) *H*_*0*_ there are no small study effects, *pOR* pooled odds ratio, *pHR* pooled hazard ratio

**Fig. 6:**
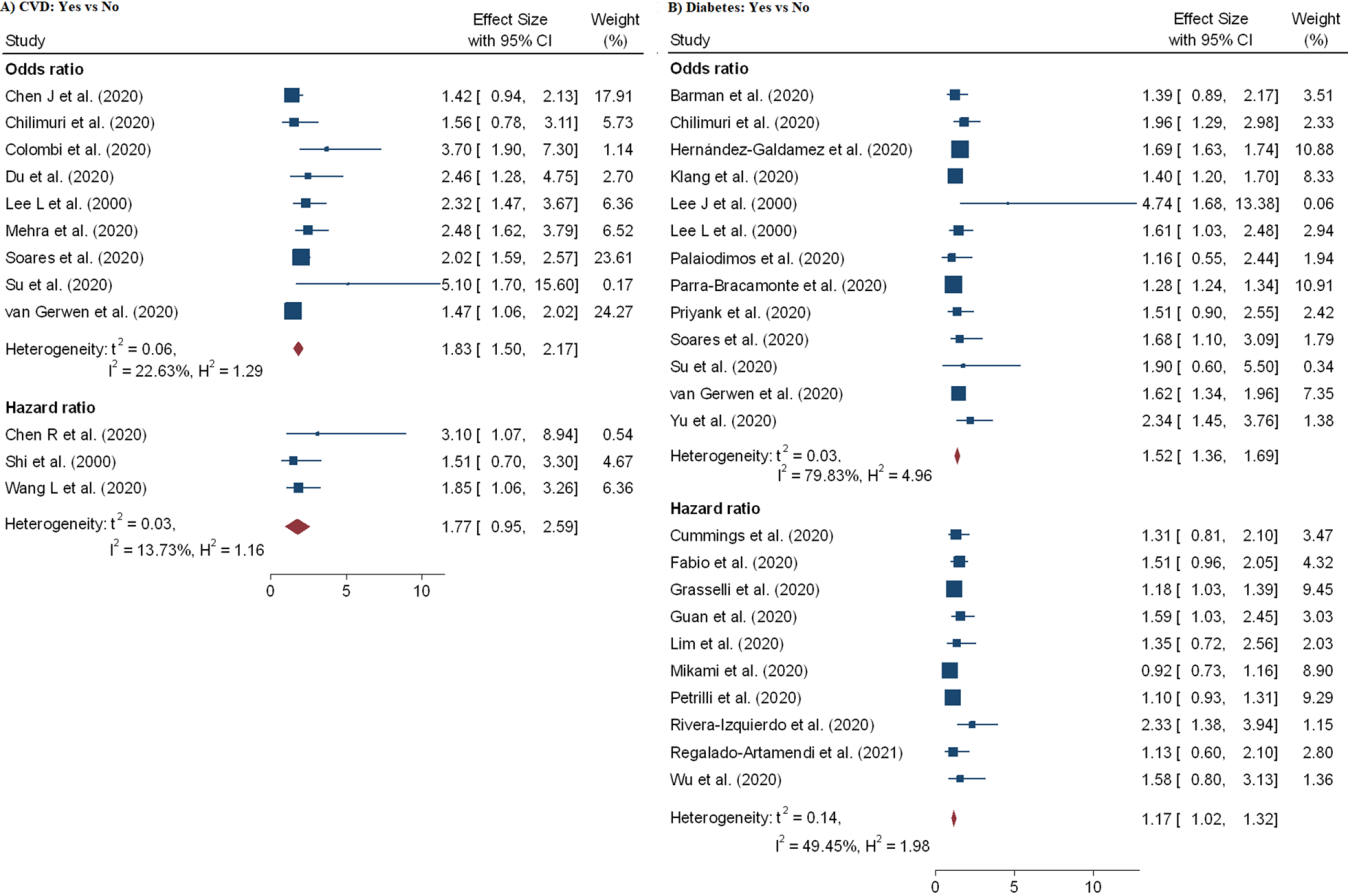
Forest plot showing the estimate for the effects of CVDs and diabetes on COVID-19 mortality

**Fig. 7:**
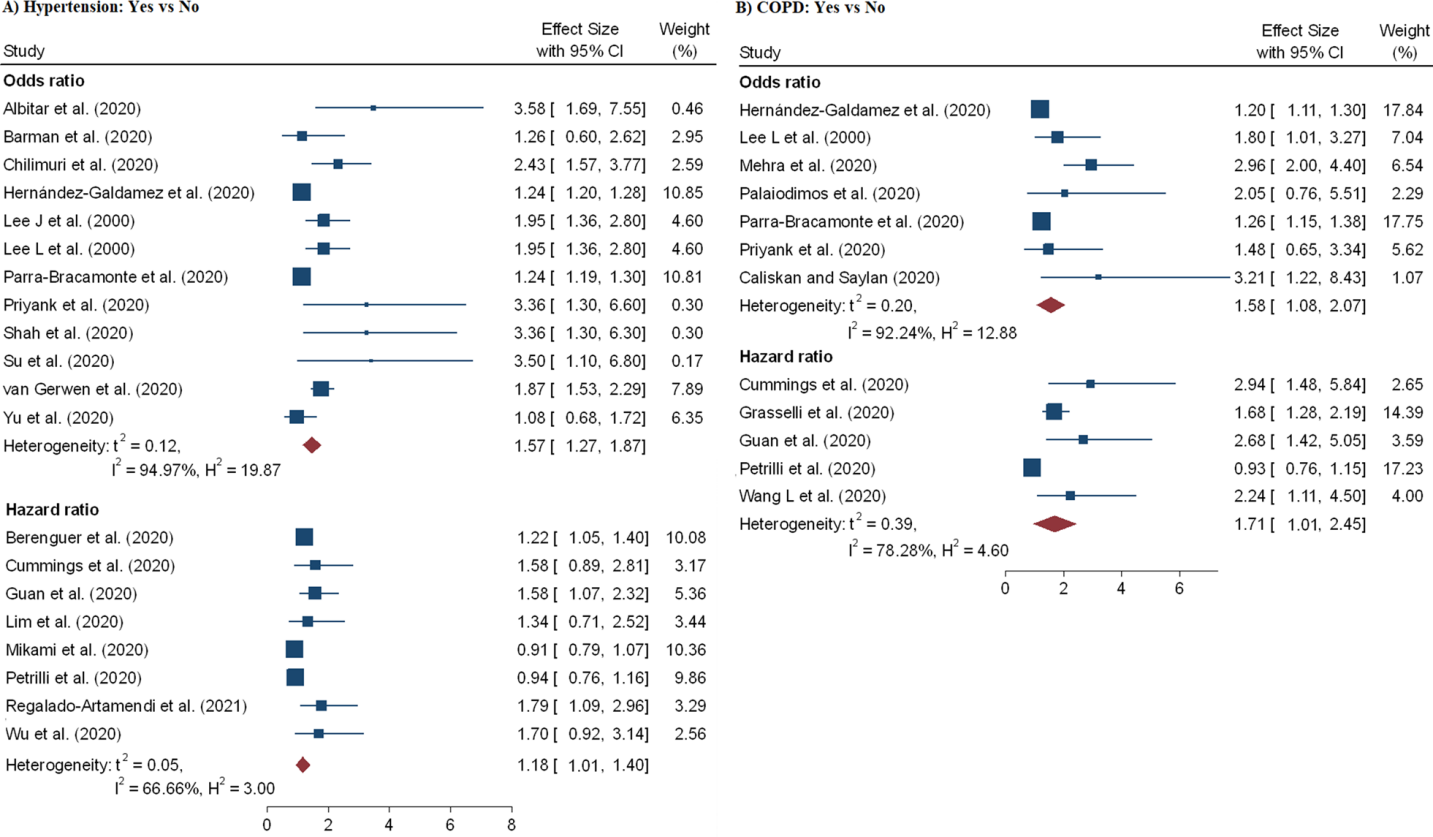
Forest plot showing the estimate for the effects of hypertension and COPD on COVID-19 mortality

**Fig. 8:**
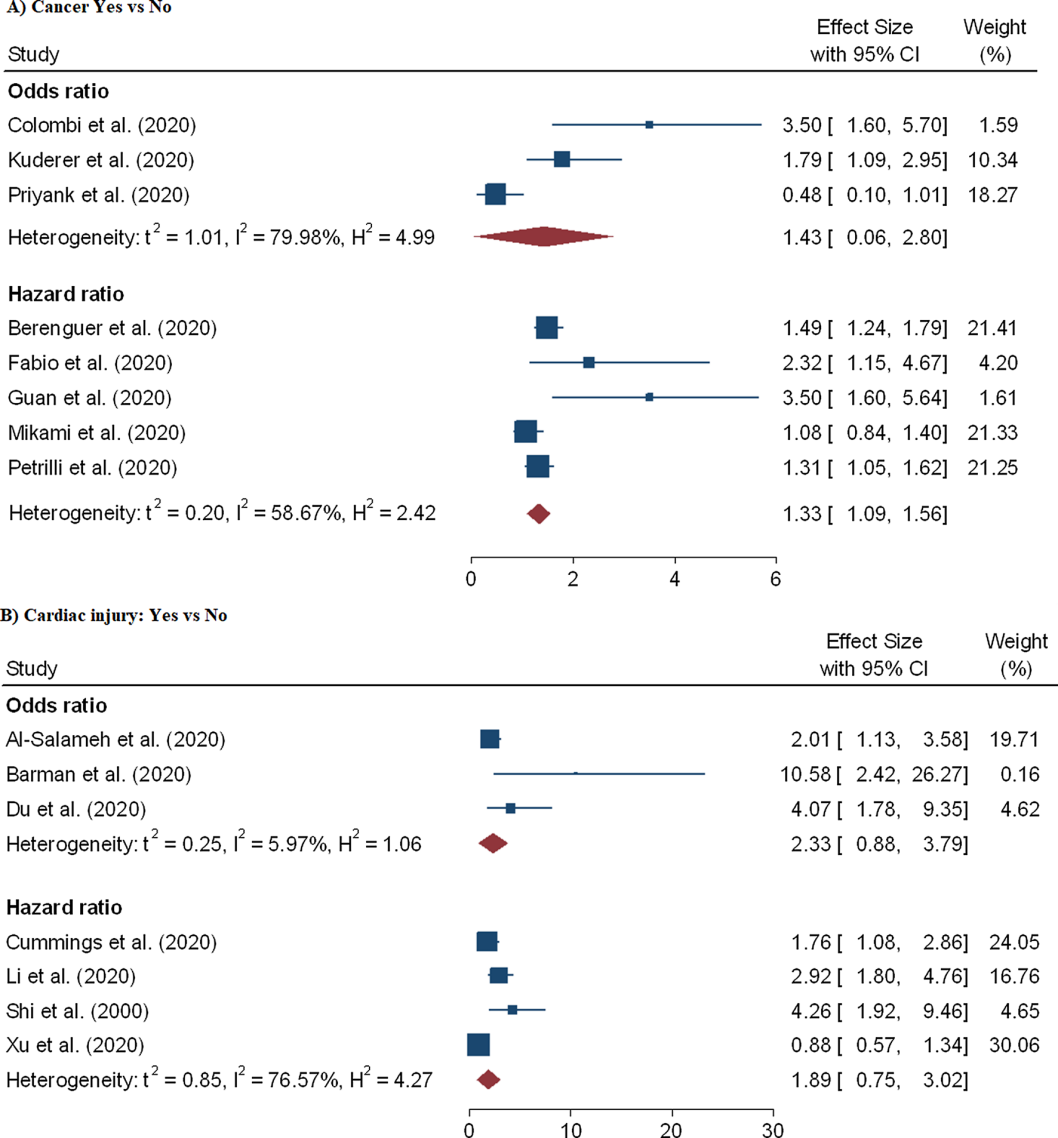
Forest plot showing the estimate for the effects of cancer and cardiac injury on COVID-19 mortality

A total of 60 effect sizes of comorbidities were extracted from 34 studies [3, 9, 11, 12, 15, 18, 21–24, 27, 28, 31, 39, 40, 44, 48, 50–52, 54–57, 67, 69, 71–73] with a total of 407, 638 patients and 32, 465 death. The association between diabetes and in-hospital mortality are displayed in Table 2 and Fig. 6B. We noted that mortality among hospitalized COVID-19 patients with diabetes was higher compared to the patients without diabetes aOR = 1.52 (95% CI 1.36–1.69) and aHR = 1.17 (95% CI 1.02–1.32). Likewise, risk of mortality among hospitalized COVID-19 patients is highly influenced by patients with COPD (pOR = 1.58; 95% CI 1.08–2.02; pHR = 1.71; 95% CI 1.01–1.40) (Fig. 7B), hypertension (pOR = 1.57; 95% CI 1.27–1.87; pHR = 1.18; 95% CI 1.01–2.07) (Fig. 7A), CVD (pOR = 1.83; 95% CI 1.50–2.17) (Fig. 6A) and cancer (pHR = 1.33; 95% CI 1.09–1.56) (Fig. 8A).

**Fig. 9:**
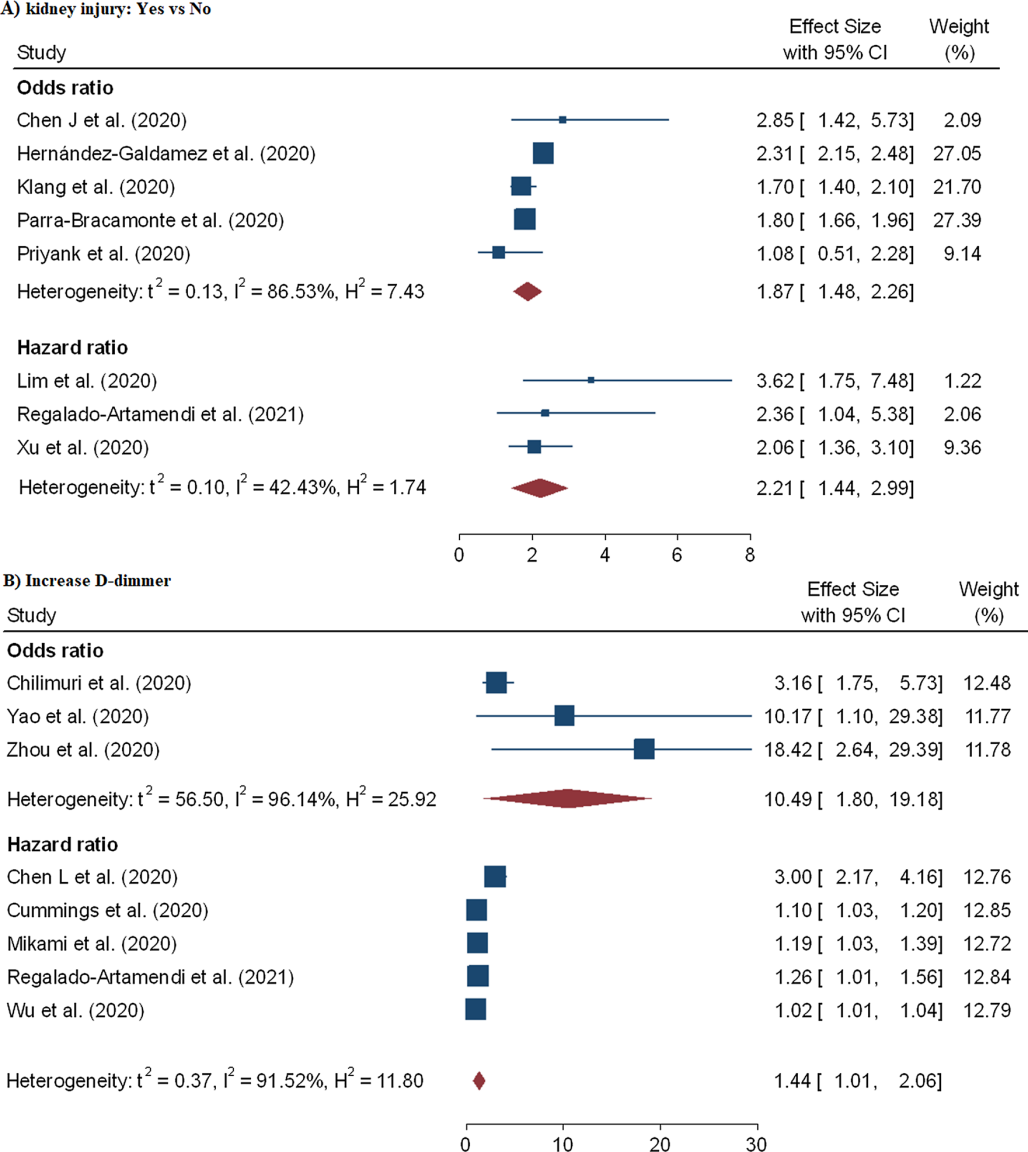
Forest plot showing the estimate for the effects of acute kidney injury, and D-dimer on COVID-19 mortality

In the meta-analysis of eight effect sizes from eight studies [19, 26, 30, 34, 39, 40, 48, 74], we noted that a significant positive association between acute kidney injury and COVID-19 mortality and the pooled OR and HR were 1.87 (95% CI 1.48–2.26) and 2.21 (95% CI 1.44–2.99), respectively (Fig. 9A). But acute cardiac injury association with COVID-19 fatality was not found to be significant (pOR = 2.33; 95% CI 0.88–3.79; pHR = 1.89; 95% CI 0.75–3.02) (Fig. 8B). Furthermore, the combined effect sizes from six studies [10, 17, 18, 32, 45, 53] revealed a significant association between increase D-dimer and coronaviruses mortality (pOR = 10.49; 95% CI 1.80–19.18) and (pHR = 1.44; 95% CI 1.01–2.06) (Fig. 9B).

**Quality Assessment**

The Newcastle–Ottawa score of the included studies was 7–9, and the quality of the articles was evaluated as high (Table 3).

**Table 3:** Risk of bias assessment of 42 studies included in the meta-analysis by the Newcastle–Ottawa Scale

Authors (year)	Selection (4)				Comparability of Cohorts (2)		Outcome (3)			Total
	Representativeness of exposed cohort	Selection of non-exposed cohort	Ascertainment of exposure	Demonstration that outcome of interest was not present at the start of study	Study control for age and sex	Additional factors; controlled for ≥ 2 variables including comorbidities	Assessment of outcome	Was follow-up long enough for outcomes to occur	Adequacy of follow up of cohorts	
Albitar et al. [51]	1	1	1	1	2		1	1	1	9
Al‐Salameh et al. [52]	1	1	1	1	0		1	1	1	7
Barman et al. [50]	1	1	1	1	2		1	1	1	9
Berenguer et al. [49]	1	1	1	1	2		1	1	1	9
Caliskan and Saylan [82]	1	1	1	1	1		1	1	1	8
Chen, et al. [46]	1	1	1	1	0		1	1	1	7
Chen et al. [47]	1	1	1	1	1		1	1	1	8
Chen et al. [48]	1	1	1	1	2		1	1	1	9
Chilimuri et al. [45]	1	1	1	1	1		1	1	1	8
Colombi et al. [55]	1	1	1	1	1		1	1	1	8
Cummings et al. [71]	1	1	1	1	1		1	1	1	8
Du et al. [44]	1	1	1	1	1		1	1	1	8
Fabio et al. [43]	1	1	1	1	1		1	1	1	8
Grasselli et al. [42]	1	1	1	1	1		1	1	1	8
Guan et al. [41]	1	1	1	1	1		1	1	1	8
Hernández-Galdamez et al. [40]	1	1	1	1	1		1	1	1	8
Klang et al. [39]	1	1	1	1	1		1	1	1	8
Kuderer et al. [38]	1	1	1	1	1		1	1	1	8
Lee et al. [37]	1	1	1	1	1		1	1	1	8
Lee et al.[36]	1	1	1	1	2		1	1	1	9
Li et al. [35]	1	1	1	1	1		1	1	1	8
Lim et al. [34]	1	1	1	1	1		1	1	1	8
Mehra et al. [33]	1	1	1	1	1		1	1	1	8
Mikami et al. [32]	1	1	1	1	1		1	1	1	8
Palaiodimos et al. [31]	1	1	1	1	2		1	1	1	9
Parra-Bracamonte et al. [30]	1	1	1	1	2		1	1	1	9
Petrilli et al. [29]	1	1	1	1	2		1	1	1	9
Pettit et al. [28]	1	1	1	1	0		1	1	1	7
Price-Haywood et al. [27]	1	1	1	1	1		1	1	1	8
Priyank et al. [26]	1	1	1	1	2		1	1	1	9
Regalado-Artamendi et al. [74]	1	1	1	1	2		1	1	1	9
Rivera-Izquierdo et al. [25]	1	1	1	1	1		1	1	1	8
Shah et al. [24]	1	1	1	1	2		1	1	1	9
Shi et al. [23]	1	1	1	1	1		1	1	1	8
Soares et al. [22]	1	1	1	1	0		1	1	1	7
Su et al. [54]	1	1	1	1	1		1	1	1	8
van Gerwen et al. [21]	1	1	1	1	1		1	1	1	8
Wang et al. [20]	1	1	1	1	1		1	1	1	8
Wu et al. [53]	1	1	1	1	1		1	1	1	8
Xu et al. [19]	1	1	1	1	1		1	1	1	8
Yao et al. [18]	1	1	1	1	1		1	1	1	8
Yu et al. [83]	1	1	1	1	2		1	1	1	9
Zhou et al. [17]	1	1	1	1	0		1	1	1	7

### Sensitivity analysis, publication bias, and heterogeneity

**Fig. 10:**
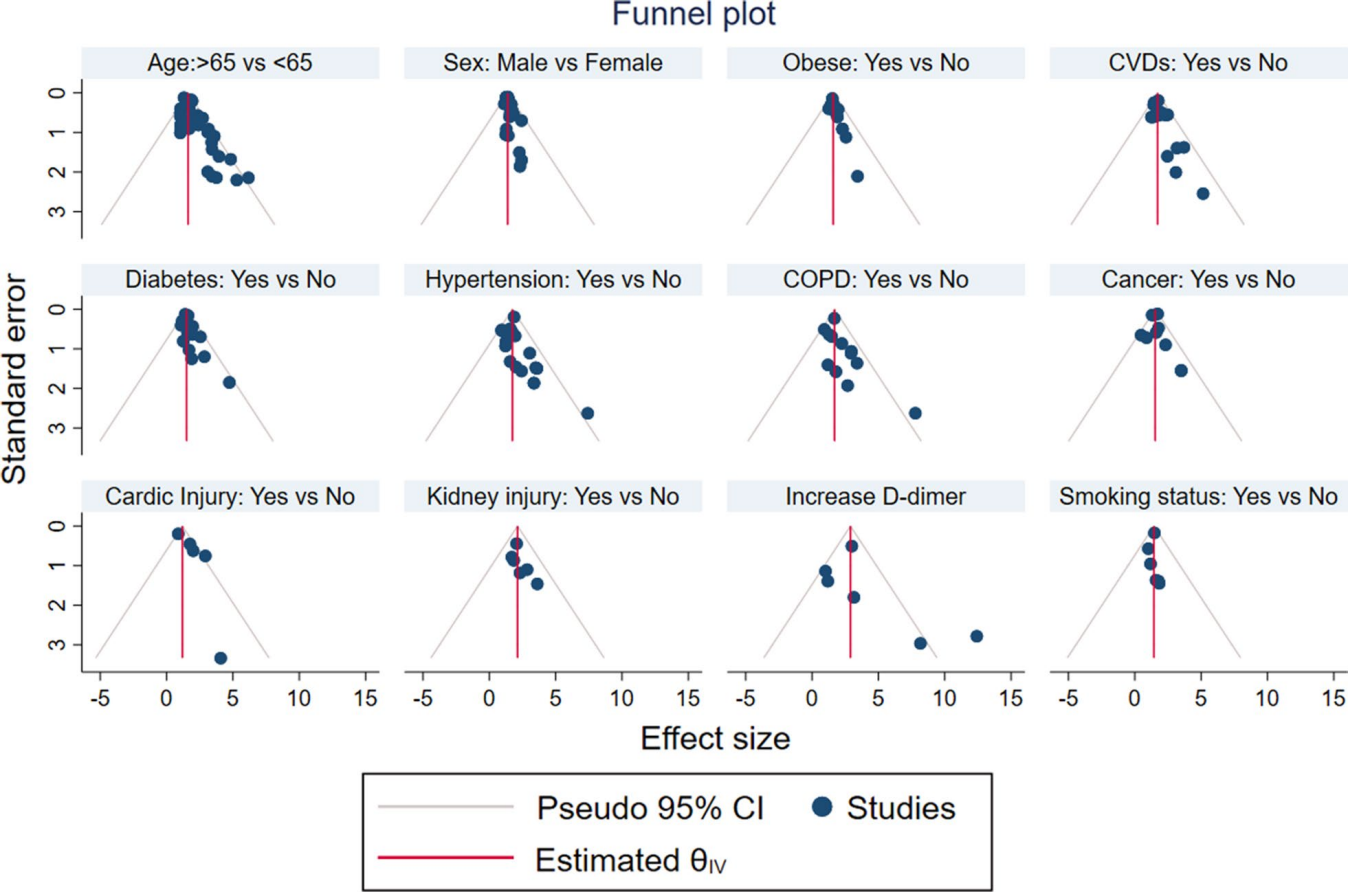
Funnel plot for publication bias of effect of comorbidities, age, smoking status, obesity, acute kidney injury, gender, and D-dimer on fatal outcome of COVID-19

The *I*^*2*^ statistics for gender, smoking status, obesity, CVDs, COPD, hypertension, cardiac injury, cancer, age, and d-dimer, had shown heterogeneities among the considered studies. From the sensitivity analysis, we noted that the overall estimates of comorbidities, gender, age, smoking status, obesity, acute kidney injury, and D-dimer on the fatal outcome of coronavirus, did not depend on a single study. Funnel plots were plotted for the included studies in the meta-analysis, which suggested that there is no noticeable bias in the studies of our meta-analysis (Fig. 10). Besides, Begg’s correlation rank and egger’s regression failed to show significant publication bias (see Table 2).

## Discussion

The meta-analysis of currently available regional and national reports of patients with coronavirus infection highlights the effect of complications, comorbidities, and demographic variables on mortality of coronavirus. These results have important clinical implications such as on the clinical management and specific preventive measures of coronavirus patients. Our study is by far the largest meta-analysis on COVID-19 fatality study in terms of size and coverage of complications, comorbidities, behavioural and demographic risk factors.

We found that smoking was significantly associated with the risk of mortality in coronavirus. Such a result was also reported [64] in a limited scale meta-analysis study. Accordingly, perhaps it is a high time step up effort to advocate the danger of smoking as well as an intervention to stop smoking to reduce the overall disease burden.

Reportedly old age was significantly associated with MERS-Cov [66] and SARS [68] mortality. Likewise, our finding showed a significant association of old age with coronavirus mortality. A plausible reason for this might be some age-related chronic medical conditions and/or lower immunity level [57]. In addition, ageing affects CD4 + T cells, CD8 + T cells, B cells functions [75]. This age-related reduction in T cells and B cells clonal diversity is associated with impaired responses to viral infections such as influenza [76] and the excess production of type 2 cytokines could lead to prolonged pro-inflammatory immune responses and therefore perhaps contribute to poor outcomes [62].

Female with coronavirus have lower rates of hospitalization and mortality than male [77]. The results of our meta-analysis also showed that men seems to be a risk factor for COVID-19 mortality. Sex differences in both the adaptive and innate immune system have been reported previously and may account for the women advantage in coronavirus. Within the adaptive immune system, men have lower numbers of CD8 + T cell [78], CD4 + T cell [79] and decreased B cell production compared to women [79]. Moreover, since some important immune regulatory genes are located on the X chromosome, women patients might be advantaged due to a higher expression TLR7 [72]. Our systematic review result also confirmed that obesity was associated with death in coronavirus patients. Indeed previously limited scale meta-analysis study [70] had also shown the same findings.

From our systematic review, we found that diabetes, CVDs, COPD, hypertension, and acute kidney injury were the significant risk for COVID-19 mortality. These factors were also reported as the coronavirus risk factor by CDC and WHO. With regard to patients’ COPD status and COVID-19 mortality association, studies [9] have argued that COPD patients with COVID-19 showed higher rates of hospitalization and mortality. This could be due to viral infections in COPD patients increase systemic inflammation with the slow recovery of reported symptoms [80]. In addition to the influence of coronavirus, COPD patients have various comorbidities, some of which are associated with an increased risk of hospitalization [69].

Diabetes also contributes to more severe COVID-19 and higher rates of mortality [81]. Our analysis also showed that mortality among hospitalized COVID-19 patients with diabetes was higher compared to the patients without diabetes. Thus, patients with diabetes and COVID-19 often need invasive ventilation care and need intensive care unit (ICU) due to their likelihood of developing Acute Respiratory Distress Syndrome (ARDS) [73]. Another two small systematic reviews, by [67, 73] also suggested that diabetes is a determinant of severity and mortality of COVID-19 patients.

Having a high D-dimer has shown a significantly increased odds of mortality. Previous study [59] had also shown that a high level of D-dimer increases severe infection and risk of mortality. In addition a study in China [17] have shown that rising D-dimer levels during the course of hospitalization are associated with the worst long-term outcomes. Therefore, using D-dimer levels as a surrogate marker for disease severity, especially, in coronavirus patients who cannot get dedicated imaging might be beneficial.

### Study limitations

Although this systematic review presented pooled estimate from 42 studies across 13 geographical locations and may be considered broadly representative of the pandemic, our study has a few limitations. First, high heterogeneity could be found. This may relate to large variation in the sample size among studies (98–211,003 patients) and the study designs. Second, the literature on coronavirus continues to accumulate, new information and new papers published each day; therefore, our study cannot be considered as exhaustive. Finally, the sample size of some included studies was very small which might not recognize the possible factors that affects COVID-19 mortality.

## Conclusion

Our study indicated a consistent and statistically significant effect of chronic comorbidities, complications, and demographic variables including acute kidney injury, COPD, diabetes, hypertension, CVDs, cancer, increased D-dimer, male gender, older age, current smoker, and obesity on the fatal outcome of COVID-19. Urgent public health interventions should be carefully tailored and implemented on those susceptible groups to reduce the risk of mortality in patients with COVID‐19 and, then, the risk of major complications. An intensive and regular follow-up is required to detect early occurrences of clinical conditions.

## Data Availability

The dataset used and analyzed during the current study is available from the corresponding author on reasonable request.

## Abbreviations

ARDS: Acute respiratory distress syndrome
COVID-19: Coronavirus infection pneumonia 2019
MERS: Middle East respiratory syndrome
pHR: Pooled hazard-ratio
pOR: Pooled odds-ratio
PRISMA-P: Preferred reporting Items for systematic reviews and meta-analyses protocols
SARS-CoV-2: Severe acute respiratory syndrome coronavirus 2
